# Relationship Between Noninvasive Assessment of Lung Fluid Volume and Invasively Measured Cardiac Hemodynamics

**DOI:** 10.1161/JAHA.118.009175

**Published:** 2018-11-14

**Authors:** Nir Uriel, Gabriel Sayer, Teruhiko Imamura, Daniel Rodgers, Gene Kim, Jayant Raikhelkar, Nitasha Sarswat, Sara Kalantari, Ben Chung, Ann Nguyen, Daniel Burkhoff, Aharon Abbo

**Affiliations:** ^1^ Department of Cardiology University of Chicago IL; ^2^ Cardiovascular Research Foundation and Columbia University New York NY; ^3^ Sensible Medical Innovations Ltd Netanya Israel

**Keywords:** fluid management, heart failure, noninvasive lung fluid volume, pulmonary arterial wedge pressure, Heart Failure

## Abstract

**Background:**

Right heart catheterization is the gold standard in clinical practice for the assessment of cardiovascular hemodynamics, but it is an invasive procedure requiring expertise in both insertion and reading. Remote dielectric sensing (ReDS) is a noninvasive electromagnetic‐based technology intended to quantify lung fluid content.

**Methods and Results:**

In this prospective single‐center study, ReDS readings were obtained in supine position just before right heart catheterization procedure in patients with heart failure. Agreement between ReDS and pulmonary artery wedge pressure (PAWP) was analyzed. Of all, 139 patients with heart failure received hemodynamic assessment and ReDS measurement. A good correlation was found between ReDS and PAWP measurement (*r*=0.492, *P*<0.001). Receiver operating characteristic analysis of the ability to identify a PAWP ≥18 mm Hg resulted in a ReDS cutoff value of 34%, with an area under the curve of 0.848, a sensitivity of 90.7%, and a specificity of 77.1%. Overall, ReDS <34% carries a high negative predictive value of 94.9%.

**Conclusions:**

Lung fluid content, as measured by ReDS, correlates well with PAWP. The high sensitivity and specificity and especially the high negative predictive value make ReDS a reliable noninvasive tool at the point of care, to rule out elevated PAWP in patients with heart failure and to help with medical management of patients with heart failure. Further studies are warranted to compare this tool with existing tests and to relate the findings to the clinical outcomes.

**Clinical Perspective**

**What Is New?**

- We demonstrated the relationship between the remote dielectric sensing technology and invasively measured pulmonary artery wedge pressure.
- Remote dielectric sensing <34% had a high negative predictive value of 94.9% to exclude patients with pulmonary artery wedge pressure >17 mm Hg.

**What Are the Clinical Implications?**

- Remote dielectric sensing technology may be a reliable noninvasive tool to rule out ambulatory patients with elevated pulmonary artery wedge pressure.

The heart failure (HF) epidemic is increasing, with >23 million patients worldwide and 6 million people in the United States.^1^ Despite numerous attempts at containment, the cost of caring for patients with HF remains unacceptably high.^2^, ^3^ Multiple methods have been developed with the goal of reducing the high burden of hospital readmissions, including daily weights and telephone monitoring systems. However, the only approach that has been shown to be effective in reducing HF readmissions has been medical management guided by ambulatory monitoring of pulmonary artery (PA) pressure.^4^, ^5^

Dyspnea is a common symptom that can be attributed to HF, lung disease, anemia, and other conditions. In patients with HF, dyspnea is the result of salt and water retention as a consequence of the increased neurohormonal activity.^6^, ^7^ When left untreated, this leads to elevated pulmonary pressure, the sensation of shortness of breath, and, ultimately, overt pulmonary edema. PA wedge pressure (PAWP), such as provided episodically with right heart catheterization (RHC) or estimated continuously with an implantable PA pressure sensor (eg, CardioMEMS; Abbott), is considered the most reliable means for clinical evaluation of volume status. Early detection of increases of PA pressure before symptoms triggering adjustment of medical therapy (compliance with diet and medical therapies and/or adjustment of diuretic and afterload reducing agent doses) has been shown to abate the progression of symptoms and avoid the need for urgent care.^5^ However, because of the invasive nature, relatively high initial costs, challenges related to reimbursement for the procedure, and long‐term follow‐up, the adoption of this technology into clinical practice has been unexpectedly slow.^8^

A reliable, safe, and noninvasive tool for the assessment of pulmonary fluid status could replace the need for an invasive approach and would be of great value in the long‐term management of patients with HF, in providing an actionable measure that triggers HF‐specific therapy to patients evaluated in urgent care settings because of shortness of breath. Besides a better safety profile, a single noninvasive device could be used in the management of many patients cost‐effectively.

Accordingly, the purpose of the current study was to evaluate the relationship between remote dielectric sensing (ReDS) assessment of lung fluid volume and invasive hemodynamic assessment during RHC.

## Methods

The data, analytic methods, and study materials will be made available to other researchers for purposes of reproducing the results or replicating the procedure from the corresponding author on reasonable request. This was a single‐center, prospective, observational study performed in patients scheduled for an RHC on the basis of clinical need. The University of Chicago Institutional Review Board approved this study, and all patients signed informed consent before the enrollment.

### ReDS System

**Figure 1:**
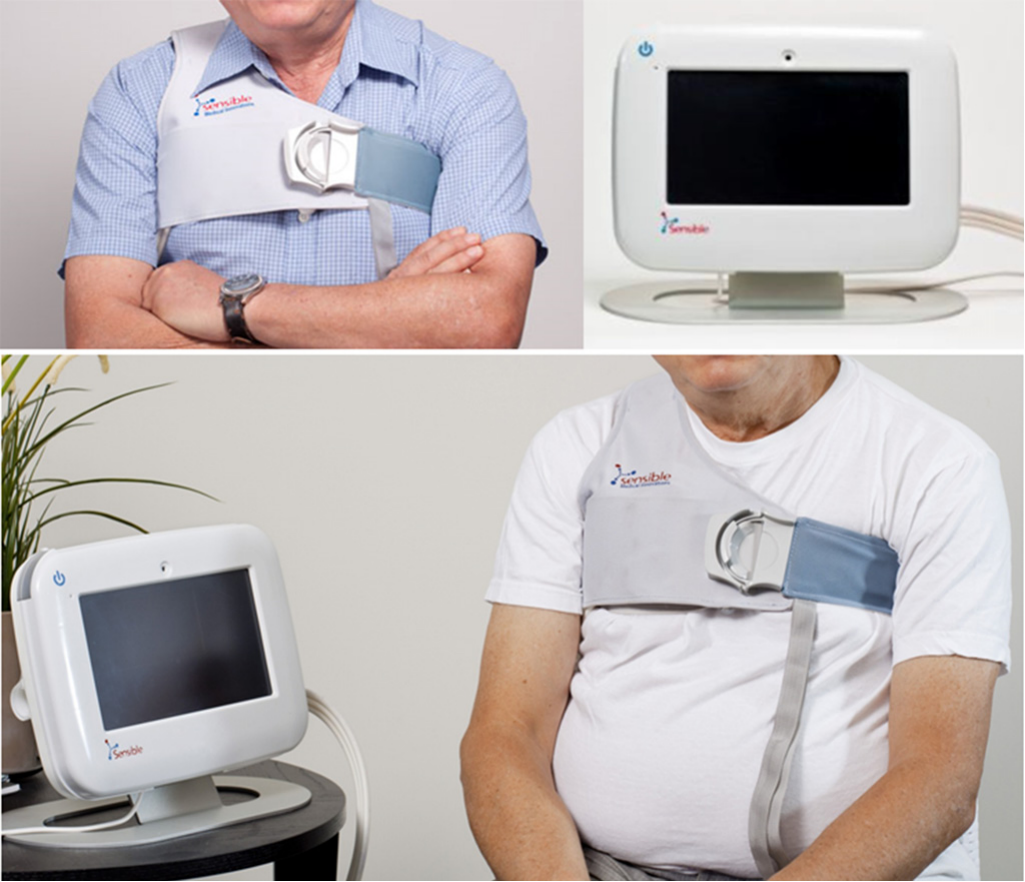
Remote dielectric sensing system: a vest and monitor to show data.

ReDS technology has been described in detail previously.^9^, ^10^, ^11^ In brief, ReDS uses low‐power electromagnetic signals emitted between 2 sensors (1 each on the anterior and posterior body surfaces) embedded in a wearable vest (Figure 1, Video S1, and YouTube channel: https://www.youtube.com/channel/UCeTy3Ifw980KCHd5_hcnKEQ). The analyzed signal reflects the dielectric properties of the portion of the lung between the sensors. The dielectric coefficient of a material is represented by a frequency‐dependent complex number describing its interaction with electromagnetic energy, including the degrees of absorption, reflection, and transmission of the energy. Because water has a high dielectric coefficient and air has a low dielectric constant, the dielectric coefficient of a tissue is determined predominantly by its fluid content. For example, healthy fat tissue, which has low fluid content, is characterized by a relatively low dielectric coefficient. Blood is relatively rich in water and is characterized by a higher dielectric coefficient. The measurement takes 90 seconds to obtain. The overall procedure to complete the ReDS measurement takes within 5 minutes. We can obtain ReDS data just by imputing patient information and pushing a start button. The normal range for the ReDS value is from 20% to 35%.

### Study Population

All consecutive patients with HF, which was diagnosed by Framingham criteria, who underwent RHC (between September 2016 and February 2017) as part of their standard of care (excluding patients with mechanical circulatory support systems) were enrolled in this study, unless they met any of the following exclusion criteria:

1 Body habitus out of range because of ≥1 of the following:
  a Height <155 cm or >190 cm or
  b Body mass index of <22 kg/m^2^ or >36 kg/m^2^.
2 Physical deformity of the thorax or lesion that may prevent proper vest application or adjustment.
3 Illness or condition that may be aggravated or cause significant discomfort by the application of the vest (eg, rib fractures, with or without flail chest, and severe osteoporosis).
4 Congenital heart malformations or intrathoracic mass that would affect the right lung anatomy (dextrocardia and lung carcinoma).
5 Pacemaker generator on the right side of the chest.
6 Vulnerable populations, such as those unable to provide informed consent and women who are pregnant or lactating.

Patients being catheterized after heart transplantation were also included.

After informed consent, baseline demographic data were collected. ReDS system readings were obtained in supine position just before transport into the catheterization laboratory for the RHC, where standard invasive hemodynamic testing was performed. In addition to quantifying mean values of central venous pressure (CVP) and PAWP obtained at the end of the expiratory phase, we also quantified large v‐waves, which were defined as a v‐wave with a magnitude of 7 mm Hg above mean PAWP. All hemodynamic parameters were measured automatically and then overread independently by 2 qualified investigators (G.S. and G.K.). When differences between values provided by the 2 investigators were within 3 mm Hg, the value of the first investigator was used. When the difference was >3 mm Hg, the one that a third independent investigator read was used as a final value. All investigators were blinded to ReDS readings and to each other. Interobserver variability in each hemodynamic variable was assessed by the Ebel intraclass correlation coefficient.

### Statistical Analyses

The primary end point of this study was an assessment of the correlation between invasive PAWP and noninvasive ReDS system measurement in patients with HF. A test for normality of data distribution was conducted using a Shapiro‐Wilk test. The PAWP‐ReDS correlation assessment was performed via Pearson's correlation coefficient.

A sensitivity and specificity analysis was performed using a receiver operating characteristic analysis to determine the ReDS value that most accurately identified whether PAWP was >18 or <15 mm Hg, a clinically meaningful cutoff value for adjustment of medical therapy. We also assessed the influence of CVP on ReDS measurement by dividing patients into 4 groups: normal CVP and PAWP, elevated PAWP with normal CVP, elevated CVP with normal PAWP, and elevated PAWP and CVP. Comparison of the ReDS values in these groups was performed with analysis of variance with a post hoc Tukey's test. For the assessment of association of variables, including ReDS measurement on PAWP, univariate and multivariate linear regression analyses were performed. Variables significant in the univariate analyses were enrolled into the multivariate analyses to construct an ideal model. In repeated analyses, paired data were compared by using the Wilcoxon signed‐rank test considering the small sample size.

All calculations were performed in SPSS Statistics 22 software (SPSS Inc, Chicago, IL), and 2‐sided *P*<0.05 was considered significant.

## Results

### Baseline Characteristics

Of all, 154 patients were screened, and 139 patients (age, 55.4±13.3 years; 100 men; and body mass index, 28.1±4.6 kg/m^2^) met inclusion criteria and were enrolled in this study. Fifteen patients with a body mass index >36.0 kg/m^2^ were excluded, and 40% of subjects were post–heart transplant. Baseline characteristics of the study participants are summarized in Table 1.

**Table 1:** Baseline Characteristics

Characteristics	All (N=139)	HF Group (N=83)	HTx Group (N=56)	*P* Value
Age, y	55.4±13.3	54.7±13.1	56.6±13.5	0.41
Body mass index, kg/m^2^	28.1±4.6	28.2±4.5	28.0±4.8	0.87
Sex (male)	100 (72)	54 (65)	46 (82)	0.021^a^
Race				
White	79 (57)	46 (55)	33 (59)	0.41
Black	49 (35)	30 (36)	19 (34)	0.47
Hispanic	7 (5)	5 (6)	2 (4)	0.52
Others	4 (3)	2 (2)	2 (4)	0.69
Echocardiography (N=116)				
Left ventricular diastolic diameter, cm	5.38±1.33	5.90±1.33	4.54±0.85	<0.001^b^
Left ventricular ejection fraction, %	45.0±18.7	35.9±17.5	58.7±10.4	<0.001^b^
Left ventricular ejection fraction >45%	67 (58)	20 (30)	47 (94)	<0.001^a^
Comorbidity				
Diabetes mellitus	50 (36)	26 (31)	24 (43)	0.11
Hypertension	88 (63)	48 (58)	40 (71)	0.073
Peripheral artery disease	5 (4)	2 (2)	3 (5)	0.36
Atrial fibrillation	38 (27)	29 (35)	9 (16)	0.011^a^
History of ventricular tachyarrhythmia	35 (25)	20 (24)	15 (27)	0.43
Chronic obstructive pulmonary disease	8 (6)	6 (7)	2 (4)	0.36

Data are given as mean±SD or number (percentage). HF indicates heart failure; HTx, heart transplantation.

a *P*<0.05 between the HF and HTx groups, by χ^2^ test or Fisher's exact test, as appropriate.

b *P*<0.05, by unpaired *t* test.

### ReDS and Hemodynamic Parameters

ReDS, PAWP, and CVP were all normally distributed. Interrater reliabilities of CVP, mean PA pressure, and PAWP were 0.968, 0.926, and 0.946, respectively.

**Figure 2:**
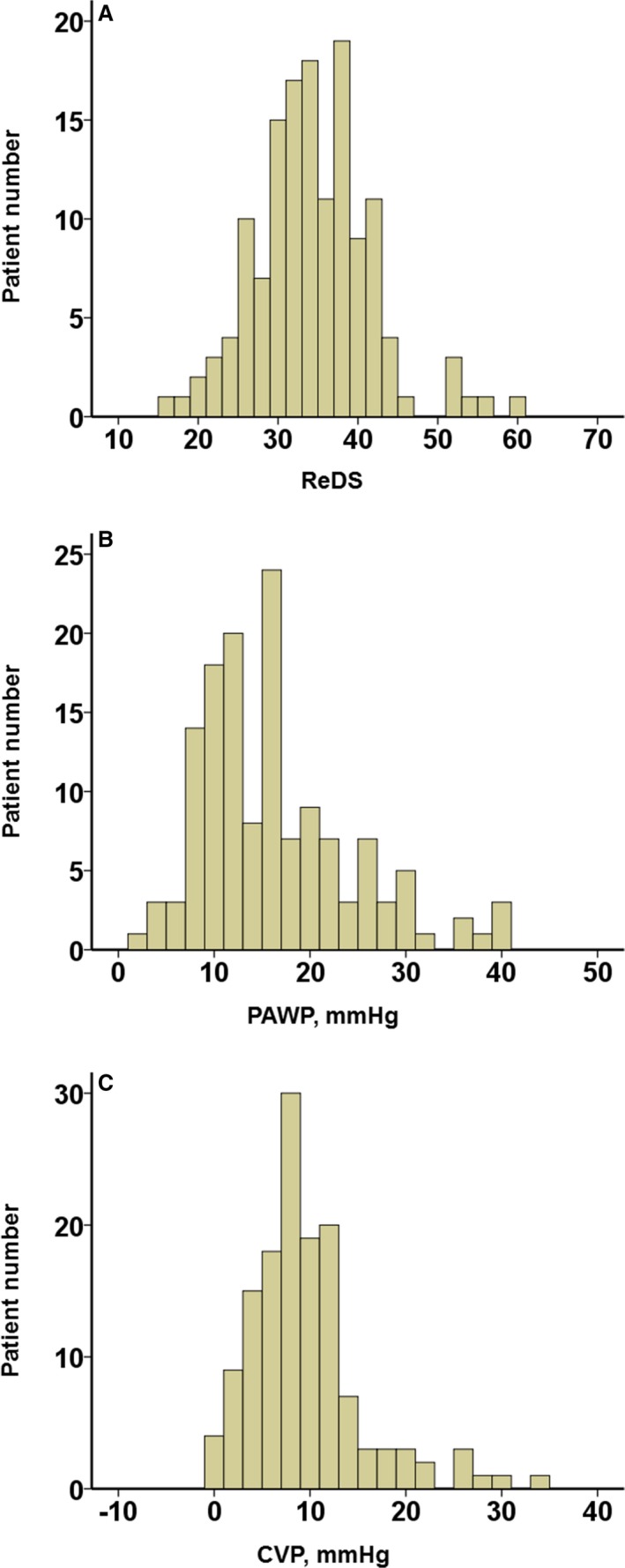
Distributions of remote dielectric sensing (ReDS;** A**), pulmonary artery wedge pressure (PAWP;** B**), and central venous pressure (CVP; **C**).

The ReDS measurement averaged 33.9±7.3% and ranged from 16% to 59% (Figure 2A). ReDS measurements greater than the upper limit of normal (ie, ≥35%) were present in 61 patients (44%). PAWP had a mean value of 15.8±8.0 mm Hg. PAWP was >18 mm Hg in 43 patients (31%) (Figure 2B). Mean CVP was 9.1±6.0 mm Hg. Thirty‐four patients (24%) had CVP >12 mm Hg (Figure 2C).

**Figure 3:**
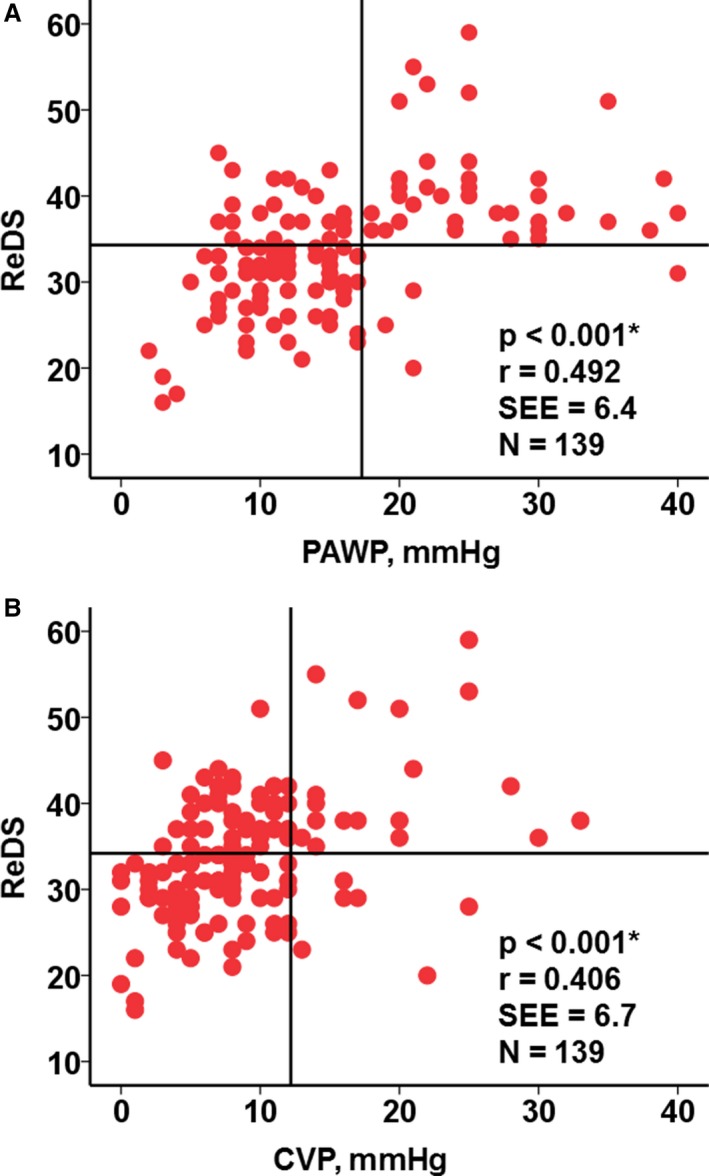
Relationships between remote dielectric sensing (ReDS) and pulmonary artery wedge pressure (PAWP;** A**) and between ReDS and central venous pressure (CVP; **B**). *P<0.001 by Pearson's correlation coefficient.

There was a positive correlation between ReDS measurement and PAWP (*r*=0.492, *P*<0.001), and the linear regression was PAWP=−2.392+0.538×ReDS (Figure 3A). CVP also had a positive correlation with ReDS measurement (Figure 3B; *r*=0.406, *P*<0.001). The relationships between the similar variables (ReDS versus PAWP and ReDS versus CVP), standardized per SD, are also shown in Figure S1.

When dichotomizing patients into high or normal PAWP (with 18 mm Hg as a cutoff) and high or normal ReDS (with 34% as the cutoff), we found that 113 of 139 patients (81.3%) had concordant PAWP and ReDS measurements (ie, PAWP <18 mm Hg and ReDS <35% or PAWP ≥18 mm Hg and ReDS ≥35%), whereas only 26 of 139 patients (18.7%) had discordant measurements (ie, 4 of 26 had PAWP ≥18 mm Hg but ReDS <35% and 22 of 26 had PAWP <18 mm Hg and ReDS ≥35%).

**Figure 4:**
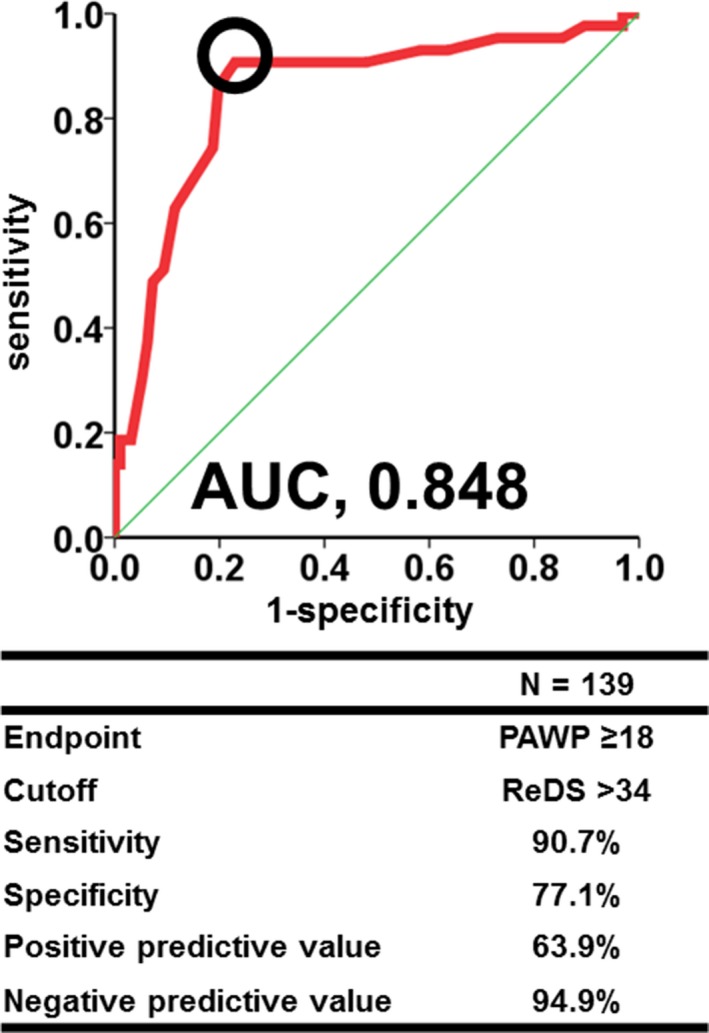
A receiver operating characteristic analysis for prediction of high pulmonary artery wedge pressure (PAWP) from remote dielectric sensing (ReDS). AUC indicates area under the curve.

Receiver operating characteristic analysis of the ability to use ReDS to identify a PAWP ≥18 mm Hg resulted in a ReDS cutoff value of 34% (Figure 4), with an area under the curve of 0.848, a sensitivity of 90.7%, and a specificity of 77.1%. Overall, ReDS <34% carries a high negative predictive value of 94.9%. When we use PAWP >15 mm Hg as an end point, which is a cutoff of pulmonary hypertension attributable to left heart disease,^12^ predictability of ReDS for elevated PAWP was similarly high compared with the results when using a cutoff of 18 mm Hg (Table S1).

**Figure 5:**
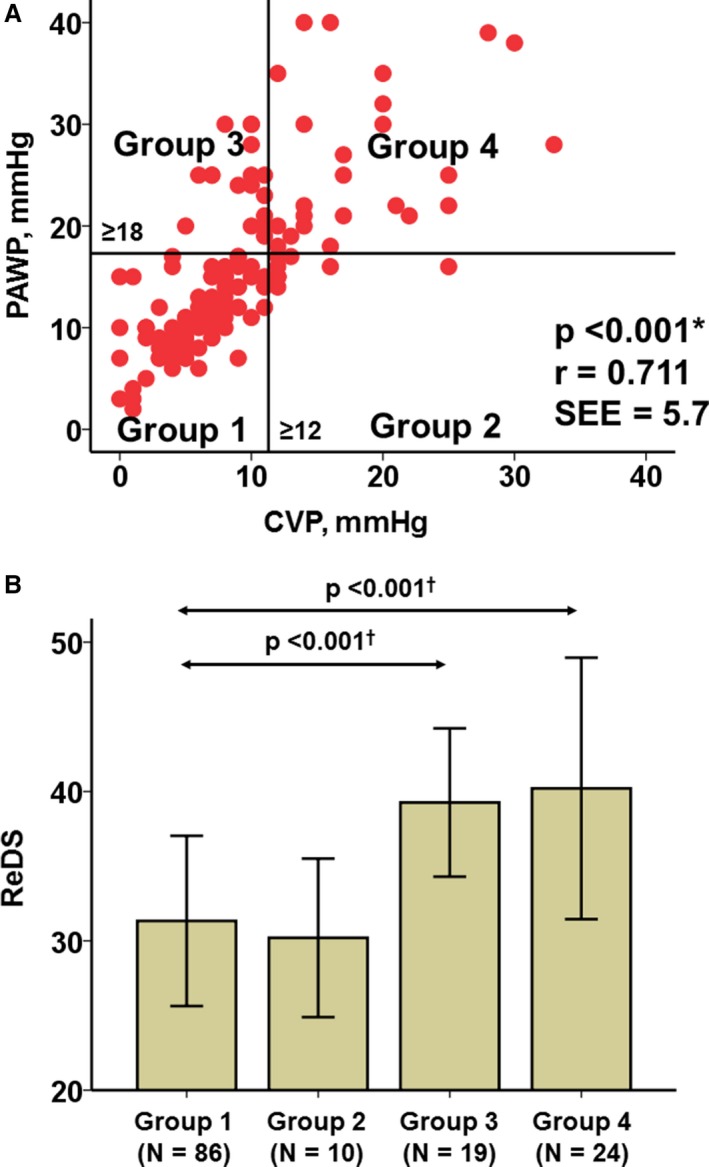
Relationship between central venous pressure (CVP) and pulmonary artery wedge pressure (PAWP; **A**) and remote dielectric sensing (ReDS) levels at each group stratified by CVP and PAWP levels (**B**). *P<0.001 by Pearson's correlation coefficient. ^†^ *P*<0.001 by post hoc Tukey's test and analysis of variance compared with group 1.

We next examined the impact of CVP and PAWP on ReDS measurement after separating patients into 1 of 4 groups on the basis of normal or elevated PAWP and CVP values (Figure 5A). PAWP and CVP were highly correlated (*r*=0.711, *P*<0.001). Group 1 patients were those with normal CVP (<12 mm Hg) and normal PAWP (<18 mm Hg), group 2 patients had high CVP and normal PAWP, group 3 patients had normal CVP and high PAWP, and group 4 patients had elevated CVP and PAWP. ReDS was significantly higher in groups with elevated PAWP (ie, groups 3 and 4) compared with groups with PAWP <18 mm Hg (ie, groups 1 and 2) (Figure 5B). On the basis of this, we can conclude that PAWP, not CVP, was the main determinant of ReDS measurement.

Twenty‐two patients had ReDS ≥35% (suggesting a hypervolemic state) and PAWP <18 mm Hg. The clinical characteristics of those patients were similar to the overall patient population, except that they had a significantly higher body mass index (30.9±4.2 versus 27.5±4.5 kg/m^2^; *P*<0.001) and more patients were in the hospital (10/22 [45.5%] versus 33/117 [28.2%]; *P*=0.090).

Finally, a univariate linear regression analysis that included clinical and hemodynamic variables was performed to determine factors that affected PAWP. The only variables identified to correlate with PAWP (with *P*<0.05) were included in a subsequent multivariate analysis, which was followed by a second multivariate analysis with parameters that continued to show significance at *P*<0.05. Only ReDS, mean PA pressure, and CVP were found to be related to PAWP (Table 2).

**Table 2:** Linear Regression Analyses Among All Clinical Variables for High PAWP

Variable	Univariable		First Multivariable		Second Multivariable	
	B	*P* Value	β	*P* Value	β	*P* Value
CVP, mm Hg	0.946	<0.001^a^	0.295	<0.001^a^	0.294	<0.001^a^
Mean PAP, mm Hg	0.634	<0.001^a^	0.475	<0.001^a^	0.484	<0.001^a^
CI, L/min per m^2^	−4.298	<0.001^a^	−0.482	0.34	…	…
Large V wave^b^	2.276	0.32	…	…	…	…
ReDS	0.538	<0.001^a^	0.161	<0.001^a^	0.167	0.001^a^

CI indicates cardiac index; CVP, central venous pressure; PAP, pulmonary artery pressure; PAWP, pulmonary artery wedge pressure; ReDS, remote dielectric sensing.

a *P*<0.05, by linear regression analyses.

b (V wave−PAWP) >7 mm Hg. Variables significant in the first multivariate analysis were enrolled into the second multivariate analysis.

### Consecutive Measurements

Of the total cohort, 38 patients had repeated RHC. Changes in PAWP and ReDS between the first and the second measurements were shown in Figure S2. In general, changes in PAWP correlated mildly with changes in the ReDS reading (*P*=0.022, *r*=0.370).

Of 22 patients demonstrating a discordance between ReDS and PAWP (with ReDS ≥35% and PAWP <18 mm Hg), 7 had repeated RHC during the study period, and second sets of measurements of ReDS were taken (Figure S3). Of them, 5 of 7 were in the hospital at their first measurement, and all patients, except for 1, had been discharged and were at ambulatory situation before the second measurements. PAWP remained unchanged between the first and second tests (13.9±1.5 versus 13.6±3.5 mm Hg; *P*=0.60), whereas ReDS decreased significantly from 42.9±5.9% to 33.9±3.9% (*P*=0.018). Consistent with this observation, paired data from these 7 patients showed the downward movement of the PAWP‐ReDS relationship (Figure S2), with 5 of the 7 points moving in a concordant manner (ie, decreased PAWP associated with a decrease of ReDS).

### Subgroup Analysis

Transplant patients were included in this study to enrich the overall cohort with patients who were more likely to have normal hemodynamics, as demonstrated in Table 1. Results summarized in Table 3 show that PAWPs and CVPs were significantly lower in transplant than nontransplant patients (*P*<0.05 for both). Despite the differences in pretest probability, sensitivity, specificity, and negative predictive value for the ReDS, measurement of predicting a PAWP ≥18 mm Hg was similar in the 2 cohorts.

**Table 3:** Central Hemodynamics and Comparison of Predictability of ReDS for PAWP ≥18 mm Hg at a Cutoff of 35%

Variable	HF Group (N=83)	HTx Group (N=56)
CVP, mm Hg	10.5±6.9	7.1±3.7^a^
PAWP, mm Hg	17.9±9.1	12.8±4.7^a^
Sensitivity, %	89.2	100.0
Specificity, %	69.6	84.0
Positive predictive value, %	70.2	42.9
Negative predictive value, %	88.9	100.0

CVP indicates central venous pressure; HF, heart failure; HTx, heart transplantation; PAWP, pulmonary artery wedge pressure; ReDS, remote dielectric sensing.

a *P*<0.001, by unpaired *t* test.

## Discussion

We evaluated the correlation between ReDS assessment of lung fluid volume and invasive measurement of PAWP in patients undergoing RHC for any indications. Our main findings are as follows: (1) A good correlation exists between ReDS and PAWP. (2) ReDS has high sensitivity and specificity to detect abnormally high PAWP that would trigger changes in medical therapy. (3) ReDS reading in the normal range has a high negative predictive value of (95%) in ensuring the PAWP is <18 mm Hg.

The current available noninvasive tools for the HF practitioner to assess fluid congestion include physical examination, chest x‐ray, and B‐type natriuretic peptide (BNP). Both physical examination and chest x‐ray are prone to operator experience, subjective interpretation, and basic limitations, which renders them relatively unreliable.^13^, ^14^, ^15^, ^16^, ^17^, ^18^ BNP and NT‐proBNP (N‐terminal pro‐BNP) are useful to support the diagnoses or exclusion of HF in patients with dyspnea and have shown value in risk stratification.^19^, ^20^, ^21^ However, their performance as a quantitative parameter in relation to the extent of fluid overload is limited. Moreover, clinical studies have yielded conflicting results in establishing them as HF management tools.^22^, ^23^

A reliable, safe, easy‐to evaluate, and noninvasive tool for the assessment of pulmonary fluid status could replace the need for an invasive test or permanent implantable device and would be of great value in both clinical and economic terms in the long‐term management of ambulatory patients with HF by providing an actionable measure that can trigger HF‐specific therapies.

HF practitioners could benefit from an HF‐related vital sign, such as ReDS, much like blood pressure or glucose for systemic hypertension and diabetes mellitus, respectively. ReDS technology is an actionable day‐to‐day parameter that is directly related to fluid volume, according to which the HF treatment can be titered.^8^, ^24^, ^25^ The results of this study demonstrated high negative predictive value of ReDS (95%) and suggested that ReDS can fill this unmet clinical need.

In 15.8% of patients, ReDS measurements indicated that patients’ lung fluid was higher than normal, yet PAWP was normal. In a smaller number of patients (2.8%), ReDS was normal, yet PAWP was elevated. There are some potential reasons for such discordance. First, a perfect correlation between ReDS and PAWP is not expected, because lung fluid content and intravascular pressures are related, but they measure different properties. Extravascular volume is a complex and variable function of vascular volume, vascular tone, hematocrit, and blood oncotic pressure; the elements of time, especially in the context of changing fluid status because of volume retention, and treatments, such as diuretics, further complicate the situation.

Accordingly, several studies highlight the low expected correlation between these 2 entities.^26^, ^27^ For example, there is a documented time lag between reductions in PAWP, observed in the long‐term setting as well as of short‐term pharmacological intervention (eg, diuretic, vasodilator, or inotrope administration), and reductions in extravascular lung fluid.^28^, ^29^ Similarly, there will also be time lags between PAWP increases and extravascular fluid volume retention in the setting of short‐term fluid loading.^6^

Thus, the stability of a patient's fluid status and timing from diuretic dosing can influence the ReDS‐PAWP relationship.^30^ Consistently, we observed in 7 patients with high ReDS and normal PAWP that such elevated ReDS levels were decreased, despite persistently normal PAWP, probably because of therapeutic interventions (5 of 7 were in the hospital at the first measurements, and all except for 1 had been discharged and were at ambulatory situation before the second measurements). Most patients with elevated ReDS but low PAWP were in‐hospital patients. In other words, we observed best concordance between ReDS and PAWP (>80%) in ambulatory patients, which is the intended setting for the use of ReDS as a point‐of‐care device.

Second, depending on the chronicity of the HF state, there can be changes in pulmonary vasculature that influence flux of fluid between compartments and the equilibrium between intravascular and extravascular fluid volume.^31^, ^32^ Given all these unknowns and confounding factors, it is emphasized during long‐term outpatient follow‐up (either daily at home or episodically in the clinic) that tracking changes in fluid balance in response to interventions (eg, changes in drug therapies and medical and dietary compliance) is as important as the absolute value itself.^33^, ^34^

However, the mechanism of an elevated ReDS measurement in the presence of normal PAWP in several populations is unclear. Currently, we believe that it is important to present the data as is and continue to learn and explore more about this technology. Nevertheless, on the basis of ReDS correlation with PAWP and its high negative predictive value, ReDS measurements as a point‐of‐care assay can help guide clinical decision making, such as treatment and patient disposition, and can also exclude elevated filling pressures that would warrant hospitalization. When a patient with shortness of breath has a low ReDS, <35%, other causes should be explored. In addition, predictability of ReDS for PAWP >15 mm Hg, which is one generally accepted threshold for defining pulmonary congestion attributable to left heart disease in patients with pulmonary hypertension, was similarly high compared with the results when using a cutoff of 18 mm Hg. Among patients with pulmonary hypertension, ReDS technology may be helpful to distinguish noninvasively whether the cause is attributable to left heart disease or not.

This study enrolled all comers to the catheterization laboratory, which included in‐hospital and out‐of‐hospital patients with HF and posttransplant patients. This provided a cohort with a wide range of hemodynamic profiles with different pretest probabilities of PAWP ≥18 mm Hg (Table 3). As with any clinical tools, the pretest probability has a strong influence on positive and negative predictive values, as seen when comparing the results in the transplant populations. In both cohorts, however, there was a high negative predictive value, which is of prime importance when ruling out elevated PAWP regardless of population.

In the context of post–heart transplant management, the high negative predictive value of a normal ReDS reading can be extremely beneficial as an adjunct to other noninvasive tests to reduce the number of RHCs. As more noninvasive options to detect rejection become available, such as routine gene expression testing (eg, Allomap) and cell‐free DNA testing (eg, AlloSure), the need for routine endomyocardial biopsy will be reduced; however, these tests do not provide an assessment of filling pressures. By combining ReDS technology with noninvasive assessment of a rejection episode, we may undergo both the fluid status assessment and rejection surveillance without the need of any invasive testing.

### Study Limitations

First, this was a single‐center study. Second, there was not so strong correlation between PAWP and ReDS reading (*r*=0.492). ReDS technology cannot estimate absolute value of PAWP accurately. The great advantage of this technology is a high negative predictive value (94.9%) to exclude the patients with PAWP >17 mm Hg, particularly in an ambulatory setting. The outpatients with ReDS >34% would receive more intensive assessment (including invasive hemodynamic tests) and treatments. Nevertheless, we should understand that this high negative predictive value (94.9%) was derived from the combination of HF population (88.9%) and transplant population (100%). We cannot simply adopt our results into other background populations. Third, because this was a registry study, we did not have access to other clinical information that could have corroborated fluid status aside from PAWP. For example, physical examination findings, BNP values, chest x‐ray films, and chest computed tomography scans could have provided insights in cases of discordance between ReDS and PAWP measurements. Also, these procedures, in addition to ReDS measurement, would strengthen the utility to understand patient physiological condition via different perspectives (volume status or filling pressure) and adjust medications. Other noninvasive measurements, such as transthoracic echocardiography, lung ultrasound, and intrathoracic impedance, are also known to estimate fluid status; however, these technologies may require expertise technique or special attention for the interpretation of the obtained value. The ReDS system is the first technology that uses electromagnetic energy that travels between the sensors (chest and back) and provides the absolute value of the fluid level as a percentage. Comparison or combination with these modalities in classifying patients or predicting clinical outcomes is strongly warranted. The current study is cross‐sectional, and the trend comparing between preinterventions and postinterventions may suggest one possible explanation for the discordance between ReDS and PAWP measurements.

Another limitation is that patients with a body mass index >36 kg/m^2^ are currently excluded from the labeling for use of the ReDS device because it may falsely detect fatty tissue around the rib cage. And, to this point, the patients with a high ReDS and a low PAWP had significantly higher body mass index compared with others. Further efforts should be made to overcome this limitation.

Although the cohort is inclusive of patients with chronic lung diseases or pneumonia, it is reasonable to hypothesize that such clinical situations may lead to false‐negative or false‐positive results of ReDS readings compared with estimated PAWP. As with any test, putting the results into context of all clinical data is important for interpreting and planning action. Until further research will elaborate the relationship of body mass index and ReDS, caution is warranted in the obese patient because of possible false‐positive readings, and appropriate measures to verify the clinical diagnosis accordingly should be taken. Sequential ReDS measurement by comparing against the baseline of each patient's ReDS may overcome this limitation.

We did not perform any clinical outcomes associating with ReDS results. The SMILE study (Sensible Medical Innovations Lung fluid status monitor allows reducing readmission rate of HF patients study), which is a randomized open‐label study to assess the utility of daily ReDS measurements on HF readmission will be able to provide some insights into the role of ReDS in titrating and managing medication, although hemodynamics data will not be available. Combination with other traditional procedures, including BNP measurement, is a next concern.

## Conclusions

Lung fluid content, as measured by ReDS, correlates well with invasively measured PAWP. The high sensitivity and specificity, and especially the high negative predictive value, make ReDS a reliable noninvasive means to rule out elevated PAWP in patients with HF, with low burden to both patients and healthcare providers. This simple noninvasive test could help detect early fluid retention in patients with HF before acute decompensated HF symptoms develop, allowing caregivers to manage patients with HD in early stages of volume accumulation. Further investigations, especially longitudinal studies, are warranted.

## Sources of Funding

The registry data collection was sponsored by Sensible Medical Innovations Ltd.

## Disclosures

Imamura receives financial support from a Postdoctoral Fellowship for Research Abroad of Japan Society for the Promotion of Science. Burkhoff is a consultant to Sensible Medical Innovations Ltd. Abbo is an employee of Sensible Medical Innovations Ltd. The remaining authors have no disclosures to report.

## Supporting information


**Table S1.** Predictability of ReDS for PAWP >15 mm Hg at a Cutoff of 35
**Figure S1.** Relationships between ReDS and PAWP (**A**) and between ReDS and CVP (**B**) (variables are standardized per standard deviation).
**Figure S2.** Changes in PAWP and changes in ReDS between the first tests and the second tests.
**Figure S3.** Trend of ReDS and PAWP between the first and second measurements in 7 patients with ReDS >35 and PAWP ≥18 mm Hg at the first measurement.

**Video S1.** Explanation of how to measure ReDS. ReDS indicates remote dielectric sensing.
