# Supplementary material for: Relationship Between Noninvasive Assessment of Lung Fluid Volume and Invasively Measured Cardiac Hemodynamics
Source: J Am Heart Assoc. 2018 Nov 14;7(22):e009175. doi: 10.1161/JAHA.118.009175 (PMC6404458; doi:10.1161/JAHA.118.009175)
Supplement: Supplementary file 1 — Table S1. Predictability of ReDS for PAWP >15 mm Hg at a Cutoff of 35 Figure S1. Relationships between ReDS and PAWP (A) and between ReDS and CVP (B) (variables are standardized per standard deviation). Figure S2. Changes in PAWP and changes in ReDS between the first tests and the second tests. Figure S3. Trend of ReDS and PAWP between the first and second measurements in 7 patients with ReDS >35 and PAWP ≥18 mm Hg at the first measurement. [file JAH3-7-e009175-s001.pdf]

SUPPLEMENTAL MATERIAL

Table S1. Predictability of ReDS for PAWP >15 mmHg at a cutoff of 35.

**Sensitivity, %**	75.0%
**Specificity, %**	77.1%
**Positive predictive value, %**	68.9%
**Negative predictive value, %**	82.1%

Figure S1. Relationships between ReDS and PAWP (a) and between ReDS and CVP (b) (variables are standardized per standard deviation).

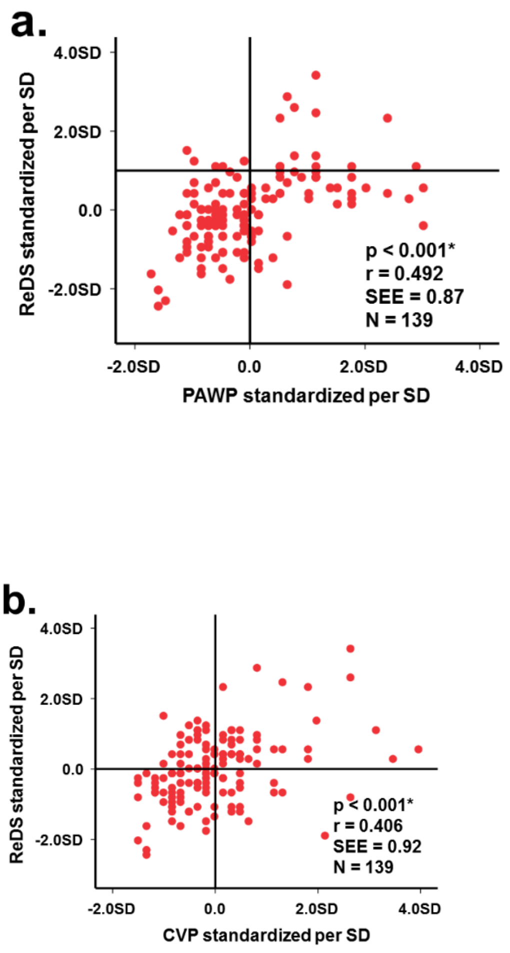

p <0.001* by Pearson's correlation coefficient.

ReDS, Remote Dielectric Sensing; PAWP, pulmonary artery wedge pressure; CVP, central venous pressure; SEE, standard error of estimate; SD standard deviation; SD, standard deviation.

Figure S2. Changes in PAWP and changes in ReDS between the first tests and the second tests.

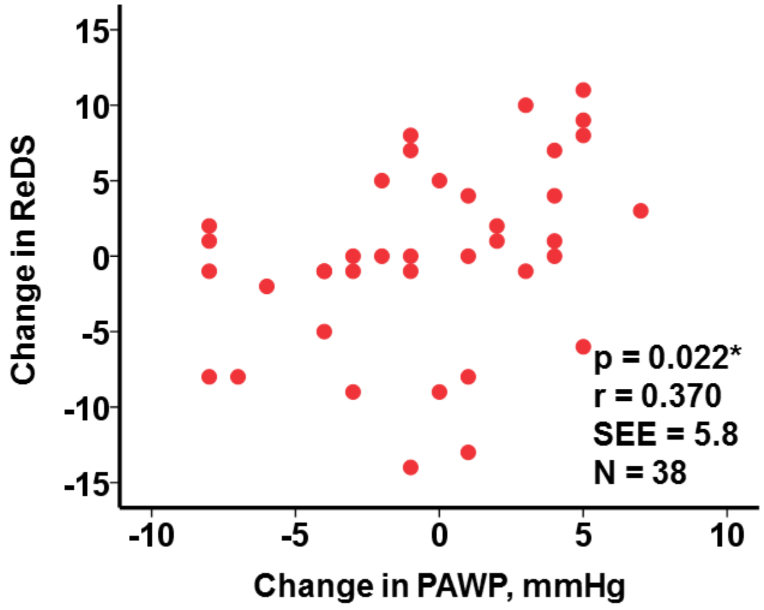

P <0.05* by Pearson's correlation coefficient.

PAWP, pulmonary artery wedge pressure; ReDS, Remote Dielectric Sensing; SEE, standard error of estimate.

Figure S3. Trend of ReDS and PAWP between the first and second measurements in 7 patients with ReDS >35 and PAWP ≥18 mmHg at the first measurement.

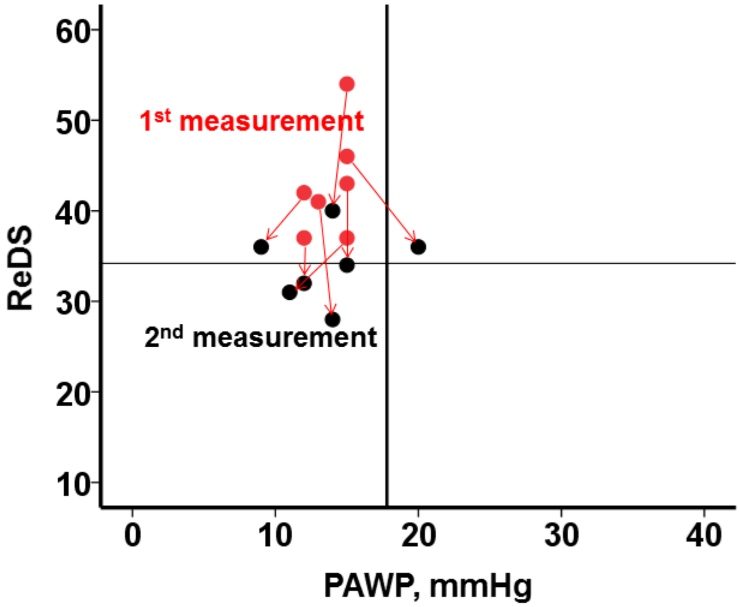

ReDS, Remote Dielectric Sensing; PAWP, pulmonary artery wedge pressure.

Supplemental Video Legend

Video S1. Explanation of how to measure ReDS. ReDS, Remote Dielectric Sensing.
